# Needs, Duties and Privileges of the Medical Profession

**Published:** 1855-07

**Authors:** 


**Needs, Duties and Privileges of the Medical Profession. By John McCall, M. D., Utica, N. Y:**

**Introductory Lecture to the Class of 1855, of the Medical Department of the University of Missouri. By J. R Allen, M. D., Prof. of Obstetrics and Diseases of Women and Children.**

Valedictory Address to the Graduates of the same Class. By Payton Spencer, M. D., Prof. of Physiology and Comparative Anatomy.

Analysis of Blandon Springs. By Professors J. L. and W. P. Riddell, of the University of La., and Prof. R. T. Brumsby, of the University of Alabama.

Thirty-Eighth Annual Report on the state of the Asylum for the Relief of Persons Deprived of the use of their Reason. Philadelphia, 1855.

The first of these pamphlets, whose titles are given above, is a miscellaneous essay on physics and metaphysics, morals and religion, as connected with medicine.

The author thinks that "Something more of Positivity is needed in respect to the brain and nervous tissues"—an opinion in which all, no doubt, will coincide. He has evidently studied the French philosophy—is a great admirer of Mons. Auguste Comte, almost worships Gall, Spursheim and Combe, and is a firm believer in the mental and physical degeneracy of the present age. It is difficult to say what the author really thinks or believes in reference to our needs, duties and privileges. The essay, however, contains many excellent sentiments and some valuable facts, which it would be well for the younger members of the profession to remember.

The address of Prof. Allen has for its theme—Man as the subject of Medical Administration. The style and manner is good, and the matter excellent. We cannot forbear giving the following closing paragraphs, which contain the author's ideas of the province of medicine.

"What then does the contemplation of man in his medical relations, if I may so express it, involve?

It embraces, as we have said, an inquiry into his whole constitution, physical, vital, intellectual and moral.

It should view him in every relation into which this compound nature may bring him—it estimates the results of their action and reactions, in numbers yet uncounted, in variety and modes of action, in health and disease, uncalculated.

It includes the observation of disease in all its manifestations; a proper appreciation of the uniting sympathies of a complicated and heterogeneous, yet harmonizing, whole—it duly weighs the recuperative powers of the system; properly estimates the auxiliary powers of art, and decides upon the time to interfere—the remedial agents to be used, and the point at which we should forbear.

Who then is equal to the task? Certainly no one fully. Our faculties are limited. All we can do is to bring every light of science to converge upon the subject, all the maxims of experience to bear upon each case, to invoke the combined indications of all appreciable surrounding circumstances, and upon bases thus derived erect the indications of cure—and then to select from the vast resources of the materia medica and other sources such remedies as are presumed from a knowledge of their action, best to fulfill the end proposed; then leave results to a higher power than our own.

Thus, and thus alone, can we fulfill the implied vows which we take in entering the profession, and thus, and thus alone, will we, as enlightened and honest practitioners, secure the only applause of true value, that of heaven and our own."

The Valedictory Address by Prof. Spence, is devoted to a consideration of the laws of life and health as developed by mortuary and statistical tables. He concludes that in some sections of our country and in England the average duration of human life has rapidly decreased in the last thirty or forty years. This has been caused mainly by the increased mortality among children. The style and views of the author may be gathered in part from the following extracts:

"God does not take little children from our midst because they are too pure for earth; for by purity alone can this temple ever be purified. Nor does he take them because fathers and mothers are too fond of them; for parent and child are magnets of his own wondrous making, that must ever be drawn to each other by a natural law, which death itself cannot repeal; though death may test its strength, and draw out the sympathies of the living in that strange, bewildering, agonizing search of the kindred spirit that has departed—of the little magnet soul that still attracts—but whence, or how, or whither, the bereaved heart knows not, but only feels its tendrils radiating and stretching out into that deep, dark void of unfathomable mystery—the home of the spirit.

But children die, because fathers and mothers, the proximate and remote progenitors have sowed the seeds of premature death in their systems, and because fathers and mothers and society have not placed them under good hygienic conditions, and fed, clothed, and watered them in obedience to the demands of health. If God sows, he will most assuredly reap. But there is no truth more evident than this, that the harvest of death among children may be and should be diminished by properly directing human efforts."

The last part of the address proposes the remedies for the evils, which are, according to the writer—

First. The awaking in the public mind of a deep and permanent interest on the subject of hygiene and medicine.

Secondly. The diffusion of more perfect knowledge concerning the laws of health, and the relations of the living organism to the external world. The proposed results of this is the prevention of a large number of diseases, and the consequent saving of many lives.

The analyses of Professors Riddell and of Prof. Brumsby, show that the Blandon springs are *alkaline and alkaline sulphuretted.* They have been a favorite resort during the summer months, and we have no doubt but that they may have a happy influence in establishing the secretions which become so often arrested by the malarious influences of the region of the Gulf of Mexico.

The report on the state of the Asylum for the relief of persons of unsound mind, shows the institution to be in a flourishing condition. During the year ending the first of April, there were admitted, forty-two; remaining on hand April 1st., 1854, fifty-seven —making in all ninety-nine patients during the year. Discharged or died, forty; remaining April 1st., 1855, fifty-nine.

Of the forty discharged, seventeen were restored, three much improved, seven stationary, eight died.

Of the fifty-nine remaining, there are restored, three; much improved, two; improved, fifteen; stationary, thirty-nine.

The provisions of the present age for the cure and treatment of the insane are comparatively good, but not equal to the wants of this unfortunate class of community. All of our Asylums are crowded. In our own State of Illinois there is an urgent demand for increased accommodations, and we are glad to know that at the last meeting of our State Society, a Committee was appointed to memorialize the Legislature on that subject, and also upon the question of making some provision for Idiots. J.

